# Aflatoxin Contamination of Commercial Maize Products during an Outbreak of Acute Aflatoxicosis in Eastern and Central Kenya

**DOI:** 10.1289/ehp.7998

**Published:** 2005-08-10

**Authors:** Lauren Lewis, Mary Onsongo, Henry Njapau, Helen Schurz-Rogers, George Luber, Stephanie Kieszak, Jack Nyamongo, Lorraine Backer, Abdikher Mohamud Dahiye, Ambrose Misore, Kevin DeCock, Carol Rubin, the Kenya Aflatoxicosis Investigation Group

**Affiliations:** 1 National Center for Environmental Health, Centers for Disease Control and Prevention, Chamblee, Georgia, USA; 2 Foreign Agricultural Service, U.S. Department of Agriculture, Nairobi, Kenya; 3 Office of Plant and Dairy Foods, Center for Food Safety and Applied Nutrition, Food and Drug Administration, College Park, Maryland, USA; 4 Kenya National Public Health Laboratory, Nairobi, Kenya; 5 Kenya Field Epidemiology and Laboratory Training Program, and; 6 Preventive and Promotive Health, Kenya Ministry of Health, Nairobi, Kenya; 7 Centers for Disease Control and Prevention, Kenya Office, Nairobi, Kenya

**Keywords:** aflatoxicosis, aflatoxin, corn, food safety, Kenya, maize, mold, mycotoxin

## Abstract

In April 2004, one of the largest aflatoxicosis outbreaks occurred in rural Kenya, resulting in 317 cases and 125 deaths. Aflatoxin-contaminated homegrown maize was the source of the outbreak, but the extent of regional contamination and status of maize in commercial markets (market maize) were unknown. We conducted a cross-sectional survey to assess the extent of market maize contamination and evaluate the relationship between market maize aflatoxin and the aflatoxicosis outbreak. We surveyed 65 markets and 243 maize vendors and collected 350 maize products in the most affected districts. Fifty-five percent of maize products had aflatoxin levels greater than the Kenyan regulatory limit of 20 ppb, 35% had levels > 100 ppb, and 7% had levels > 1,000 ppb. Makueni, the district with the most aflatoxicosis case-patients, had significantly higher market maize aflatoxin than did Thika, the study district with fewest case-patients (geometric mean aflatoxin = 52.91 ppb vs. 7.52 ppb, *p* = 0.0004). Maize obtained from local farms in the affected area was significantly more likely to have aflatoxin levels > 20 ppb compared with maize bought from other regions of Kenya or other countries (odds ratio = 2.71; 95% confidence interval, 1.12–6.59). Contaminated homegrown maize bought from local farms in the affected area entered the distribution system, resulting in widespread aflatoxin contamination of market maize. Contaminated market maize, purchased by farmers after their homegrown supplies are exhausted, may represent a source of continued exposure to aflatoxin. Efforts to successfully interrupt exposure to aflatoxin during an outbreak must consider the potential role of the market system in sustaining exposure.

Mycotoxins are fungal metabolites that can contaminate agricultural products and threaten food safety. The Food and Agriculture Organization estimates that mycotoxins contaminate 25% of agricultural crops worldwide (Smith et al. 1994). Aflatoxins, a group of mycotoxins mainly produced by *Aspergillus flavus* and *Aspergillus parasiticus*, are of particular public health importance because of their effects on human health. Aflatoxins have both carcinogenic and hepatotoxic actions, depending on the duration and level of exposure. Chronic dietary exposure to aflatoxins is a major risk factor for hepatocellular carcinoma, particularly in areas where hepatitis B virus infection is endemic. Ingestion of higher doses of aflatoxin can result in acute aflatoxicosis, which manifests as hepatotoxicity or, in severe cases, fulminant liver failure (Fung and Clark 2004). Contamination of food supplies by these and other naturally occurring toxins is of particular concern in rural communities of developing countries (Bhat et al. 1997).

Outbreaks of acute aflatoxicosis from highly contaminated food have been documented in Kenya, India, and Thailand [Council for Agriculture Science and Technology (CAST) 2003]. In April 2004, an outbreak of acute hepatotoxicity was identified among people living in Kenya’s eastern and central provinces. Epidemiologic investigations determined that the outbreak was the result of aflatoxin poisoning from ingestion of contaminated maize (corn). As of 20 July 2004, 317 cases and 125 deaths had occurred, making this one of the largest and most severe outbreaks of acute aflatoxicosis documented worldwide [Centers for Disease Control and Prevention (CDC) 2004]. Because of the high number of cases and large geographic area involved, health officials were concerned that aflatoxin-contaminated maize might be circulating in the regional maize distribution system. To assess the potential exposure to aflatoxin through consumption of commercial maize products (market maize), we conducted a cross-sectional assessment of market maize contamination. The primary objectives of this study were to characterize the extent of aflatoxin contamination within the maize market system and to assess the relationship between market maize aflatoxin levels and the outbreak of aflatoxicosis. In addition, we sought to identify factors contributing to aflatoxin contamination of market maize.

The outbreak covered more than seven districts encompassing an area approximately 40,149 km^2^ (15,502 mi^2^). Of the 317 case-patients, 89% resided in four districts (Makueni, Kitui, Machakos, and Thika). The estimated total population of these four districts is 2.8 million (Central Bureau of Statistics 1999). Of the four districts, Makueni and Kitui were most heavily affected (representing 47% and 32% of case-patients, respectively), followed by Machakos (6% of cases) and Thika (4% of cases) (CDC 2004).

Overall, the area has a rural population that is primarily from the Akamba ethnic group. Most of the local population engages in small-scale, mixed farming that includes some livestock. Maize is the primary dietary staple and the main crop produced. At harvest, farmers store most of their maize for household consumption and sell the rest to meet other household needs. When household maize stores are exhausted, farmers purchase maize back from market vendors.

Maize is distributed through a network of rural markets. Small lots of maize from local farmers are pooled and may be combined with imported maize and redistributed. No formal records of maize sources or trade are available at this level of distribution (Oduor J, personal communication). The markets are a mixture of small, family-owned shops providing consumer goods and services, and traditional open-air markets where migrant vendors bring products to sell or trade.

The market maize assessment presented in this article is one of three complementary, epidemiologic investigations conducted in response to the aflatoxicosis outbreak. First, a descriptive epidemiologic investigation was performed. Based on hypotheses generated by the descriptive investigation, two concurrent, complementary investigations were conducted: a case–control investigation of the outbreak and the assessment of market maize. An abbreviated description of all three studies and preliminary findings were reported in the *Morbidity and Mortality Weekly Report* in September 2004 (CDC 2004).

## Materials and Methods

This study was conducted, beginning 4 June 2004, over a 3-week period during the peak of the outbreak. We collected maize samples from markets located in the four districts where 87% of the aflatoxicosis case-patients resided (Makueni, Kitui, Machakos, and Thika). We interviewed vendors and collected maize products in major agricultural markets in the districts most affected by aflatoxicosis. Markets in 5 of the 31 divisions within the four study districts were not sampled for logistical reasons.

### Market selection.

Individual agricultural markets in each district were selected for inclusion on the basis of information obtained from interviews with the district agriculture officer of each district. We created a sample of major agricultural markets that represented potential exposure to aflatoxin among all market maize consumers within the study area. Markets were selected for inclusion based on the following criteria: *a*) geographic location of the population served by the market, *b*) having an increased number of maize vendors, *c*) having a variety of maize vendor types, and *d*) holding an important position in the maize distribution system for the district.

Large government grain warehouses operated by Kenya’s National Cereals and Produce Board (NCPB) also were included in this study. The NCPB is involved in grain marketing and acts as a strategic grain reserve for food supply functions of the country, including famine relief.

### Vendor selection.

At each market surveyed, vendors were selected to create a sample that included all types of maize vendors represented within each marketplace. Vendor types were store merchant, wholesale maize distributor, small-scale miller, or open market vendor (i.e., migrant vendor who brings products to sell in an open air market). The variability of maize sold determined the number of vendors interviewed at each market. More interviews were conducted at markets with maize from a variety of sources. Maize variability was assessed based on *a*) the size of each market, *b*) variety of vendor types present, and *c*) relationship of the market to major distribution routes within the district. Vendors were systematically selected based on location within the market in order to obtain geographic distribution within the marketplace. Selected maize vendors were administered a face-to-face interview and requested to provide samples of each of their maize products for aflatoxin analysis.

### Survey instrument.

Face-to-face interviews were conducted with maize vendors at the marketplace in Kiswahili, Kikamba, or English. All vendors were administered a standard survey questionnaire. Information was collected on market location, vendor type, vendor trade practices, maize history (as could be recalled by the vendor), and vendor’s assessment of the quality (at the time of purchase) of maize products sampled.

### Maize sample collection.

Maize products were sampled from every vendor interviewed. A 1-kg sample was taken from every maize product offered by the vendor. Maize products were dried maize kernels, maize flour (commercial or locally milled), and *muthokoi* (kernels with the outer hull removed). If the vendor offered the same product from different sources (i.e., maize kernels purchased from local farmers and maize kernels from a distributor), then a 1-kg sample was taken from each.

Most of the samples were collected from 90-kg bags of maize. Multiple samples were taken from different parts of one bag or several bags belonging to one vendor and combined to produce a 1-kg sample for analysis. The maize samples were collected using the respective vendor’s sampling tools (i.e., spikes and scoops). Samples were transported and stored in paper bags. Prepackaged 1- or 2-kg bags of commercial maize flour were also collected for analysis.

### Maize sample analysis.

The samples were analyzed for total aflatoxin using a slightly modified immunoaffinity method based on Association of Official Analytic Chemists (AOAC) method 991.3 (Trucksess et al. 1991). Briefly, the whole sample was ground to pass a No. 20 sieve, and a 50-g subsample was removed for analysis. Methanol:water (80:20) solvent (100 mL) and 5 g NaCl were added to the 50-g subsample, and the mixture was blended at high speed for 1 min. The mixture was then filtered through a fluted filter paper (Whatman 2V, Whatman plc, Middlesex, UK), and the filtrate was diluted (1:4) with water and refiltered through a glass-fiber filter paper. Two milliliters of the glass-fiber filtrate was placed on an Aflatest P immunoaffinity column (VICAM, Watertown, MA, USA) and allowed to elute at 1–2 drops/sec. The column was washed two times with 5 mL water, and aflatoxin was eluted from the column with 1 mL high performance liquid chromatography (HPLC)-grade methanol. A bromine developer (1 mL) was added to the methanol extract, and the total aflatoxin concentration was read in a precalibrated VICAMSeries-4 fluorometer set at 360 nm excitation and 450 nm emission. Samples containing > 250 ppb were repeated using a 1:49 water:sample dilution ratio. The modified fluorometry method had ≥85% recovery and a 1 ppb limit of detection.

### Data analysis.

#### Vendor type variables.

Participants were classified as one or more vendor type(s) on the basis of the type of business and maize trade in which they were engaged.

#### Geographic location variables.

District-and division-level administrative boundaries for each market were used to create geographic location variables. The four districts included in this study are divided into 31 divisions: Makueni (7 divisions), Kitui (8 divisions), Machakos (10 divisions), and Thika (6 divisions). A variable was created that dichotomized divisions into those in which one or more cases of aflatoxicosis had occurred and those with no aflatoxicosis case-patients. Data on the location of aflatoxicosis case-patients were obtained from the descriptive study of the outbreak. Data collection methods for the descriptive study have been published elsewhere (CDC 2004).

#### Maize history and vendor trade variables.

Vendors were asked where the maize was grown. Maize from within the same district as the market was considered local maize; maize from outside the district where the market was located was classified as outside maize. Outside maize was further categorized as being from Loitokitok (a major import route for Tanzanian maize), Busia (a major import route for Ugandan maize), and other districts in Kenya. Participants were asked who had sold them the maize product [i.e., local farmers, a merchant, or a lorry vendor (a migrant vendor who buys and sells from a truck)]. These variables relate to the specific maize product sampled. For mixed maize from more than one source, vendors indicated all that applied. Vendors were also asked about selling practices, including who purchases their maize products (e.g., local residents, small-scale millers, or other merchants).

#### Maize type and quality variables.

The type of maize product was indicated for each sample collected. Vendors were asked whether, in their opinion, the maize had appeared completely dry at the time of purchase. Interviewers did not inquire about methods used to assess extent of dryness.

#### Maize aflatoxin concentration.

The continuous aflatoxin concentration variable represents the individual aflatoxin concentrations for each maize product collected. A dichotomous aflatoxin variable was also created using the U.S. Food and Drug Administration (FDA) and Kenya Bureau of Standards regulatory limit for aflatoxin in products for human consumption, 20 ppb (FDA 1997; Kenya Bureau of Standards 1988). Samples were dichotomized based on whether or not the aflatoxin levels were > 20 ppb.

### Data analysis and analytic methods.

Data were analyzed using SAS computer software (SAS Institute Inc., 2001). We used mixed linear models to investigate the association between the natural log of aflatoxin concentrations in maize samples and questionnaire variables. Nested random effects (i.e., divisions within districts, markets within divisions, and vendors within markets) were added to account for potential correlation among samples. We calculated least-squares means for the fixed effects specified in the models.

## Results

### Descriptive results.

We surveyed 65 markets within the four study districts. Within those markets, we interviewed 243 vendors and collected 350 maize products (Table 1). All but two vendors we approached agreed to be interviewed and provide samples.

Most (65%) vendors were store merchants, followed by open market vendors (19%), wholesale distributors (10%), and small-scale millers (3%). The most common maize products sold in the market place were maize kernels (69%), followed by *muthokoi* (18%) and maize flour (12%). Most vendors (89%) reported that their maize products were dry at the time of purchase.

During the study period (June 2004), the maize trade was primarily local. The majority (88%) of maize was locally grown, sold to vendors by local farmers (70%), and bought by local residents (88%). Of the 45 samples representing maize products from outside the local area, 30 (67%) were from Loitokitok and or Busia, and 15 (33%) were from other Kenyan districts.

Aflatoxin levels in market maize indicate widespread aflatoxin contamination. Of the 350 market maize samples collected, 192 (55%) had levels greater than the regulatory limit of 20 ppb. One hundred twenty-one (35%) of the maize samples had aflatoxin levels > 100 ppb (five times the regulatory limit), and 24 (7%) had levels > 1,000 ppb. Aflatoxin levels ranged from 1 ppb (the lower limit of detection) to values as high as 46,400 ppb. Each of the four study districts had a substantial proportion of market maize with aflatoxin levels > 20 ppb (Table 2). Makueni and Kitui districts had the highest proportions of samples, with aflatoxin levels > 20 ppb (65% and 62%, respectively), followed by 51% of maize from Machakos markets and 34% from Thika (Table 2).

Fourteen samples were collected from NCPB warehouses in Makueni, Kitui, and Machakos districts. Of the 14, 8 (57%) had levels > 20 ppb, and 6 (43%) had levels ≥100 ppb. Among NCPB warehouses, samples from the Makueni facility contained the highest levels of aflatoxin, including two that were > 1,000 ppb.

### Analytic results.

**Figure 1:**
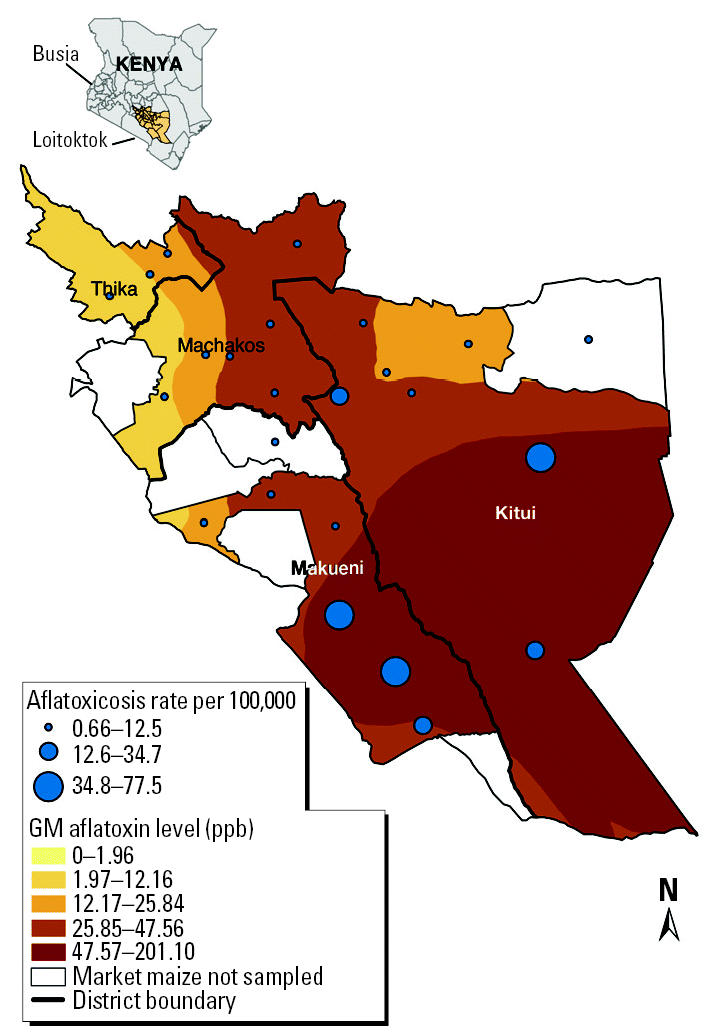
Aflatoxicosis rate and market maize aflatoxin by division in each of the four study districts. Each dot represents the rate of aflatoxicosis by division, and dots are in the center of each division (divisions are not shown).

Significant differences were found in the geometric mean (GM) of market maize aflatoxin levels between districts. These differences were consistent with the geographic distribution of aflatoxicosis cases. Makueni and Kitui, the districts with the highest number of aflatoxicosis cases, also had the highest market maize aflatoxin levels. Maize from markets in Makueni had a GM aflatoxin level greater than 2.5 times the upper acceptable regulatory limit [GM = 52.91 ppb; 95% confidence interval (CI), 27.19–103.21 ppb]. Kitui had the second highest GM aflatoxin level, followed by Machakos and Thika (Table 3, Figure 1). When aflatoxin contamination data from Makueni were compared with data from Machakos and Thika, the differences in GM aflatoxin levels were statistically significant (*p* = 0.0249 and *p* = 0.0004, respectively).

At the division level, those divisions with one or more aflatoxicosis case-patients had significantly higher aflatoxin levels in market maize than did market maize from divisions with no aflatoxicosis case-patients (GM = 27.70 ppb vs. 6.14 ppb, *p* = 0.0022).

The aflatoxin GM in locally grown market maize from within the affected area was higher than levels in market maize grown outside the local area. The difference was not, however, statistically significant (GM = 19.84 ppb vs. 9.64 ppb, *p* = 0.1748). The low aflatoxin level in maize from outside the local area primarily reflects maize from Loitokitok and Busia, major import routes from neighboring African countries. The GM aflatoxin for maize from Loitokitok and Busia was 9.14 ppb (95% CI, 3.32–25.13 ppb).

No significant differences were observed among GM aflatoxin levels of market maize based on whether or not maize was from a store merchant, open market vendor, wholesale distributor, small-scale miller, or other type of maize vendor. Aflatoxin levels did not vary significantly among the types of market maize products (i.e., maize kernels, flour, or *muthokoi*). No significant differences were seen in aflatoxin levels based on vendor selling practices or whether the maize was wet at the time of purchase.

The dichotomous aflatoxin concentration variable was analyzed to compare the odds of exposure to aflatoxin at levels > 20 ppb by market location, maize history, and vendor type. Significant differences were seen among all four study districts. The odds of exposure to aflatoxin levels > 20 ppb were more than four times higher in samples from Makueni than in samples from Thika [odds ratio (OR) = 4.29; 95% CI, 1.71–10.80]. At the division level, maize samples from markets located in divisions with aflatoxicosis case-patients were three times more likely to have maize aflatoxin levels > 20 ppb compared with samples from markets in divisions not affected by aflatoxicosis (OR = 3.13; 95% CI, 1.69–5.88). Locally grown maize from the affected area was significantly more likely to have aflatoxin levels > 20 ppb compared with maize from other regions of Kenya or imported from other countries (OR = 2.71; 95% CI, 1.12–6.59). Aflatoxin levels by type of vendor did not differ significantly.

## Discussion

Maize is the primary dietary staple in the region affected by the aflatoxicosis outbreak. Aflatoxin contamination of market maize, therefore, is an important public health concern. Our findings demonstrate widespread aflatoxin contamination of maize within the regional market distribution system. A high proportion (55%) of maize samples from markets in all four study districts had aflatoxin levels greater than the regulatory standard of 20 ppb. Twenty-four samples (7%) had exceedingly high levels (i.e., > 1,000 ppb). Thus, consumers of market maize in this area of Kenya have been at significant risk for exposure to high levels of aflatoxin.

Aflatoxin levels in market maize mirror the geographic distribution of aflatoxicosis cases associated with the outbreak. Data from this study indicate a statistically significant association between the locations of aflatoxin-contaminated market maize and cases of aflatoxicosis. However, the specific nature of this relationship cannot be inferred by findings from this study alone. We can further our understanding of how aflatoxin in market maize relates to the outbreak of aflatoxicosis by looking at findings from the complementary, case–control investigation of the outbreak (CDC 2004), in conjunction with findings from this assessment of market maize.

The case–control investigation was conducted concurrently with the market maize assessment and was limited to cases and village-matched controls in the two most affected districts (Makueni and Kitui). The case–control study showed that aflatoxicosis in the affected area was associated with eating homegrown maize and storing homegrown maize under damp conditions. The maize implicated in this outbreak was harvested in February during unseasonable, early rains. As a result, maize was stored wet under conditions conducive to mold growth. This probably led to aflatoxin contamination of farm household maize (CDC 2004).

It is likely that the contaminated, home-grown maize implicated in the outbreak entered the market distribution system when local farmers sold a portion of their farm household stores to market vendors. This information is consistent with both known trade practices in the region and reports from maize vendors and district agricultural officers during the market maize study. The vendors and agricultural officers informed us that the maize sold in the market during our study was purchased in March through May, was obtained from local farmers in the affected area, and was from the February 2004 harvest. These reports are also consistent with our findings in the market maize study that show that 88% of market maize was locally grown, and maize bought from local sources had higher aflatoxin levels than did maize bought from sources outside the affected area.

The case–control investigation also demonstrated that eating market maize was not significantly associated with aflatoxicosis in the outbreak (CDC 2004). Contaminated market maize may, however, represent a significant source of continued exposure to aflatoxin after the homegrown maize implicated in the outbreak had been consumed or discarded. Known trade practices indicate that once household stores have been depleted, local farm families are likely to buy back essentially the same contaminated maize they sold to vendors, thus continuing exposure. During the market maize study, district agricultural officers stated that local consumer demand for market maize in Makueni and Kitui was expected to increase because of depletion of farm household stores and the anticipated failure of the upcoming harvest (Oduor J, personal communication). As a result, consumer dependence on market maize was expected to be particularly high in the two districts with the highest market maize aflatoxin levels (Makueni and Kitui), thus amplifying the cycle of reexposure to aflatoxin in this population.

Our findings should be interpreted in light of some limitations. Vendors may have been reluctant to report buying and storing wet maize and following other practices known to favor fungal growth. Also, the association between the aflatoxicosis cases and market maize aflatoxin levels is ecologic and subject to ecologic fallacy. We do not, however, make causal inference based solely on ecologic data from this study. Finally, we used aflatoxin levels in maize as a surrogate for potential exposure to aflatoxins rather than measuring actual exposure using human biomarkers. Maize is, however, the dietary staple in this population, and aflatoxin levels in maize are therefore likely to provide a good indication of aflatoxin exposure (Moss 1998).

The conditions implicated in triggering this outbreak are consistent with previous reports of aflatoxicosis outbreaks. In 1981, an outbreak of aflatoxicosis from contaminated maize occurred in this same region of Kenya—the Makueni District. In both 1981 and 2004, drought and food shortages were followed by unseasonable rains during harvest, which probably favored the growth of aflatoxigenic aspergilli in household maize (Ngindu et al. 1982). The largest documented outbreak of aflatoxicosis took place in western India in 1975. This event also occurred in the context of unseasonable rains during harvest, which led to contamination of homegrown maize stored under damp conditions (Krishnamachari et al. 1975). Investigations of these previous outbreaks document the importance of unseasonable rains and improper storage of homegrown maize in aflatoxicosis outbreaks. However, they do not include documentation of potential exposure through market maize products. This study represents the only published assessment of market stores during an aflatoxicosis outbreak and the only reported investigation to explore the role of the regional market distribution system in exposure to aflatoxin.

Our assessment demonstrates that market maize represents a significant source of continued exposure to aflatoxin, long after contaminated household stores have been consumed or discarded. These data suggest that public health efforts to interrupt aflatoxin exposure during an aflatoxicosis event must include both an assessment of aflatoxin contamination within the regional market distribution system and replacement of contaminated market products.

This outbreak occurred in the context of critical regional and national food shortages resulting from prolonged drought and crop failure. Immediate response efforts have focused primarily on food replacement and relief. Some inspections of local and imported commercial products are also being conducted. Products suspected of mold contamination are being seized and replaced (Integrated Regional Information Networks 2004). To effectively prevent future outbreaks of aflatoxicosis, establishment of long-term interventions such as a comprehensive food safety program must be implemented. These interventions must target both market vendors and local farmers in order to prevent or minimize future aflatoxicosis outbreaks and reduce long-term exposure to aflatoxins.

## Figures and Tables

**Table 1:** Description of study sample [*n* (%)] by district.

Study district	Divisions	Agricultural markets	Maize vendors	Maize products
Makueni	7	16 (25)	67 (26)	96 (27)
Kitui	8	11 (17)	50 (21)	73 (21)
Machakos	10	20 (31)	66 (27)	105 (30)
Thika	6	18 (28)	60 (25)	76 (22)
Total	31	65	243	350

Values shown are the total number of markets, vendors, maize products included in the study by district and the percentage of total within the district.

**Table 2:** Distribution of aflatoxin levels in maize products collected from agricultural markets in the study districts.

			Maize aflatoxin > 20 ppb^b^ [*n* (%)]		
Study district	No. of maize products^a^	Maize aflatoxin ≤20 ppb^b^ [*n* (%)]	21–99 ppb	100–1,000 ppb	> 1,000 ppb
Makueni	91	32 (35)	12 (13)	36 (40)	11 (12)
Kitui	73	28 (38)	15 (21)	23 (32)	7 (10)
Machakos	102	50 (49)	26 (25)	23 (23)	3 (3)
Thika	76	50 (66)	13 (17)	10 (13)	3 (4)
Total	342	160 (47)	66 (19)	92 (27)	24 (7)

Values shown are the number of maize product samples with aflatoxin and the percentage of total samples within the district.

a Number of maize product samples analyzed for aflatoxin, which do not include eight samples collected but not analyzed for aflatoxin concentration.

b Acceptable upper limit for aflatoxin in grains is 20 ppb (FDA 1997; Kenya Bureau of Standards 1988).

**Table 3:** Geographic distribution by district, January through June 2004.

			Market maize aflatoxin level (ppb)	
District	No. of aflatoxicosis cases^a^	Aflatoxicosis incidence rate^b^	GM (95% CI)	Range
Makueni	129	16.7	52.91 (27.19–103.21)	1–5,400^c^
Kitui	88	17.1	35.27 (17.32–72.77)	1–25,000
Machakos	19	2.1	17.84 (9.79–32.54)	1–3,800
Thika	12	1.9	7.52 (3.83–14.78)	1–46,400
Total	233	8.2	20.53 (13.42–31.39)	1–46,400

a Total number of aflatoxin cases per district (CDC 2004).

b Incidence per 100,000 population; denominator is based on Kenya 1999 census data (Central Bureau of Statistics 1999).

c Lower limit of detection is 1 ppb.
